# Practices of maize handling and nixtamalization to reduce fungal toxin exposure in rural Guatemala

**DOI:** 10.1016/j.jafr.2024.101512

**Published:** 2024-11-12

**Authors:** Gabriela Montenegro-Benthancourt, Hannah Glesener, Olga Torres, Emeline Seegmiller, Peter Rohloff, Lee E. Voth-Gaeddert

**Affiliations:** a Center for Indigenous Health Research, Wuqu’ Kawoq|Maya Health Alliance, Tecpan, Chimaltenango, 04006, Guatemala; b Biodesign Center for Health Through Microbiomes, Arizona State University, Tempe, AZ, 85281, USA; c School for Engineering of Matter, Transport, and Energy, Arizona State University, Tempe, AZ, 85281, USA; d Laboratorio Diagnóstico Molecular, Guatemala City, 01015, Guatemala; e Centro De Investigación en Nutrición y Salud, Guatemala City, 01015, Guatemala

**Keywords:** Aflatoxin, Food safety, Public health, Maize storage, Tortillas

## Abstract

Fungal toxins in local food supplies are a critical environmental health risk to communities globally. To better characterize hypothesized toxin control points among households, we conducted household surveys across four departments (first administrative division) in Guatemala. Data gathered included maize harvesting, processing, storage, and traditional nixtamalization practices. In total, n = 33 households participated in the survey and were from 4 unique departments, 17 unique municipalities, and represented 4 different languages. The results suggested that the majority of households consumed a combination of personally cultivated and purchased maize. There was significant variation in how this maize was stored, in regards to pre-processing (kernel vs whole cob), as well as storage system type. For nixtamalization, the largest differences in practices (e.g., cooking time) were based on household size while the majority of households reported practices that aligned with previously reported best practices. Lastly, all reported maize-based food products produced by households utilized the nixtamalization process except one. Current maize handling and nixtamalization practices reported by a majority of households aligned with best practices, however, with locally tailored and culturally sensitive guidance disseminated by key stakeholders, the prevalence of best practice use among households can be improved. Further community-based research on traditional farming and nixtamalization practices can improve these recommendations.

## 1. Introduction

Food safety among staple crops is a critical aspect of child health, growth, and development [1]. In Guatemala, like many Latin American countries, maize is an important crop both from an industrial and subsistence farming perspective, making up 70 % of some communities’ diets [2]. Fungal toxins, such as aflatoxin, can contribute to significant economic losses in industrial and subsistence farming operations and poses a significant health exposure risk via human consumption of maize [3,4]. Aflatoxin B1 (AFB1) is a class 1 carcinogen that causes liver cancer with long-term exposure while shorter-term exposure effects are less well understood [5]. Identifying and strengthening critical control points and approaches to reduce fungal toxin transmission throughout regional and national maize value chains is important but complicated given numerous stakeholders and their competing priorities and obligations [6-8]. While progress for strengthening maize value chains continue at regional and national levels, households can also be further empowered to improve their subsistence maize quality and cooking practices to reduce potential exposures to dangerous fungal toxins.

From the household perspective, households have the following control points available to them to mitigate fungal toxin growth and exposure: pre-harvest crop health, post-harvest processing practices, post-harvest storage practices, maize selection in the market, purchased maize storage practices, and maize cooking (i.e., nixtamalization) [7]. There are extensive resources that provide guidance on best practices in the agricultural aspects of these control points. Garsow and colleagues provide an overview for Guatemala of these control points and ‘best--practice’ control approaches for maize cultivation, harvesting, and storage [9]. Fewer resources are available for guidance on best practices for nixtamalization. Schaarschmidt and Fauhl-Hassek provide a review of optimal parameters for nixtamalization to reduce fungal toxin bioavailability based on previous studies [10]. While they highlight significant variation in these studies, general thresholds can be extracted. For AFB1, we propose the following thresholds.

- Amount of calcium hydroxide (cal): a ratio of cal-to-water of at least ~0.33 (e.g., 1 part cal to 300 parts water; by mass)
- Maize cooking time: >40 min; especially if steep time is short (<12 h)
- Maize steeping time: >6 h, but higher is better, 8+ hours (1 h reduces AFB1 by ~50 %; while 12 h reduces AFB1 by ~90 %)
- Washing cooked maize (nixtamal): ≥1 time

While helpful, further confirmation of these thresholds is recommended. In addition, to translate these thresholds and optimize programming and engagement strategies for agricultural or community health extension agents, it is important to compare variation within current household practices to proposed best practices.

First, fungal and toxin control points within local maize value chains are available to households but often change due to seasonal factors (harvest timing, market price of maize, maize source switching, storage capacity and quality, etc.). While uniform national programming can make program implementation simpler and cheaper, creating more geographically tailored programs may be more cost-effective due to better alignment with households’ needs and implementation barriers. Therefore, understanding the variation in current farming practices across geographies and cultures is important. Second, nixtamalization processes are an important cultural practice as well as an effective process for detoxification of dangerous fungal toxins (Aflatoxin B1 and Fumonisin B1) [10]. Unfortunately, these practices have received less attention from regional or national programming efforts potentially due to the difficulties in 1) defining best practices and 2) understanding current practices and barriers-to-change among households across geographies, cultures, and economic levels.

In this paper, we report results of a household survey conducted by local agriculture extension agents across five departments in western Guatemala to identify current household practices of maize harvesting, processing, and storage as well as maize nixtamalization processes. The aim of this survey is to 1) provide critical information to implementing stakeholders on current practices and 2) demonstrate a low-cost approach to filling data gaps to improve regional and national programming to improve population health.

## 2. Materials and methods

### 2.1. Study site

Guatemala is the largest country in Central America and is home to a diverse population of more than 20 different Mayan ethnicities as well as Ladinos, Xincas, and other non-Mayan ethnicities. Maize is the staple crop and is the primary crop for subsistence farmers. The Western Highlands of Guatemala is mountainous with two primary seasons (rainy and dry) resulting in one harvest season per year while in the lowlands and coastal regions there exist two harvest seasons [11]. This dynamic often influences seasonal changes in the maize supply chain, with large portions of maize coming from coastal regions or Mexico during lean portions of the year in the Western Highlands. Mountainous, rural communities are often poorer economically and have higher rates of chronic malnutrition among children under the age of five [12]. Given the variation of cultural practices and geographies, Guatemala provides an important location to demonstrate methods to improve household support programming at the regional and national level.

#### 2.1.1. Survey

**Fig. 1.**
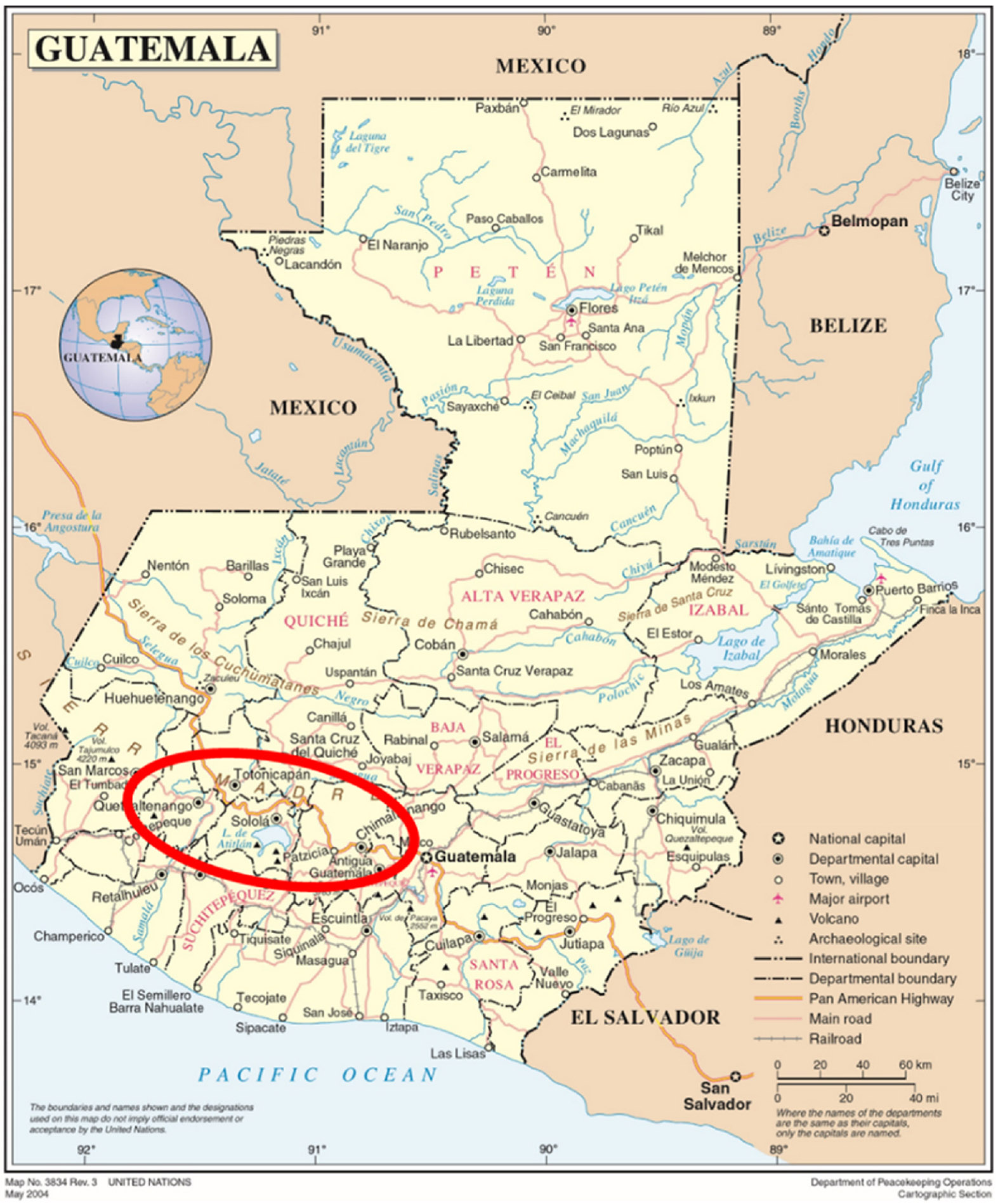
Map of Guatemala and location of the surveyed households. Graphic adapted from the United Nations Geospatial archives.

In July and August of 2023, a household survey was conducted by the agriculture extension agents of the Ministry of Agriculture, supported by the US Peace Corps and Maya Health Alliance to collect information on current practices of maize post-harvest practices, storage and nixtamalization processes (see Fig. 1). The survey was constructed in Spanish by the research team and initially piloted among a small set of households. The final survey was then administered orally by the extension agents to a household member and data recorded on paper surveys. Data was transferred to digital format for downstream analysis. Communities were selected based on a geographic gradient with the aim of incorporating four departments and at least three unique municipalities within each department. Households were selected based on a with-in community convenience sample. This sampling strategy was utilized to maximize the potential variation observed among households in maize handling practices and nixtamalization processes. The survey instrument can be found in the supplementary material in both Spanish and English. The study received a determination of exemption (non-human subjects research) from the Wuqu’ Kawoq | Maya Health Alliance Institutional Review Board (WK-2023-002).

### 2.2. Data analysis

Descriptive statistics were generated for all data collected including mean and standard deviation, median and range, and boxplots, as applicable. In addition, variables collected were stratified across geography, primary language spoken, and number of household members to visually identify potential variation driven by location, ethnicity, or economic/living situations. Appropriate bivariate statistical tests were used to evaluate significant differences between groups (e.g., geography) of the collected variables. Data were analyzed using Excel v2401 and R v4.3.1.

## 3. Results

Between July and August 2023, n = 33 households were surveyed. Households represented four different departments and 17 different municipalities. Four different languages were reported as the primary language spoken in the households (Spanish, K’iche’, Kaqchikel, and Mam) and the median number of members living in the households was six (range: 1–11). Table 1 and Table S1 presents descriptive statistics for the variables collected overall and broken down by these demographic groups.

For household maize sources utilized within the last 30 days, 70 % (23/33) of households reported consuming maize they cultivated as their primary source, while 97 % of households practiced maize cultivation to some degree. However, only 33 % of households reported just consuming their own cultivated maize, while 67 % of households either consumed a combination of cultivated and market maize or only market maize. Only 12 % of households reported consuming maize in the past 30 days from only the market. The most common harvest month in the previous year was November but ranged from September to February. Households in Chimaltenango reported later harvest dates (December–February) as compared to the other three departments.

For post-harvest processing, 64 % of households shell all their maize, while 21 % of households only shell the maize they will consume immediately (12 % fall somewhere in between; 1 household only buys maize). Of the n = 11 households that reported leaving some or all the maize on the cob for storage, 8 of 11 of those households were from Totonicapán. If households shell all their maize (n = 20), reported storage locations included silos (8/20), sacks (6/20), barrels or boxes (5/20), or spread out in a room (1/20). If the households leave the maize on the cob (n = 11), reported storage locations included a loft space, ceiling, or storeroom in the house (5/11), sacks or boxes (4/11), or traditional silo structures (2/11). If maize is purchased from the market (n = 18 reporting), reported storage locations included sacks (9/18), barrels (5/18), or silos (4/18). Of the households that reported cultivating their own maize and also purchasing maize at some point during the year (n = 18), only 11 % (2/18) reported storing this maize in a different type of storage location compared to cultivated maize. Finally, 97 % of households reported that they are intentional about keeping their maize dry in storage, but reported practices varied.

### 3.1. Maize nixtamalization and consumption

For the preparation and consumption of maize by households, 51 % of households reported using yellow maize while 49 % use white maize. This even divide generally applied across departments except in Sacatepéquez where all but one household used white maize. A similar even split was identified between households that make and consume tamales the majority of the time (51 %) as compared to tortillas (49 %). Interestingly, all households from Chimaltenango and Sacatepéquez reported making tortillas, while 81 % (17/21) of households from Quetzaltenango and Totonicapán reported making tamales. All households prepare either tortillas or tamales with nixtamalized maize.

**Fig. 2.**
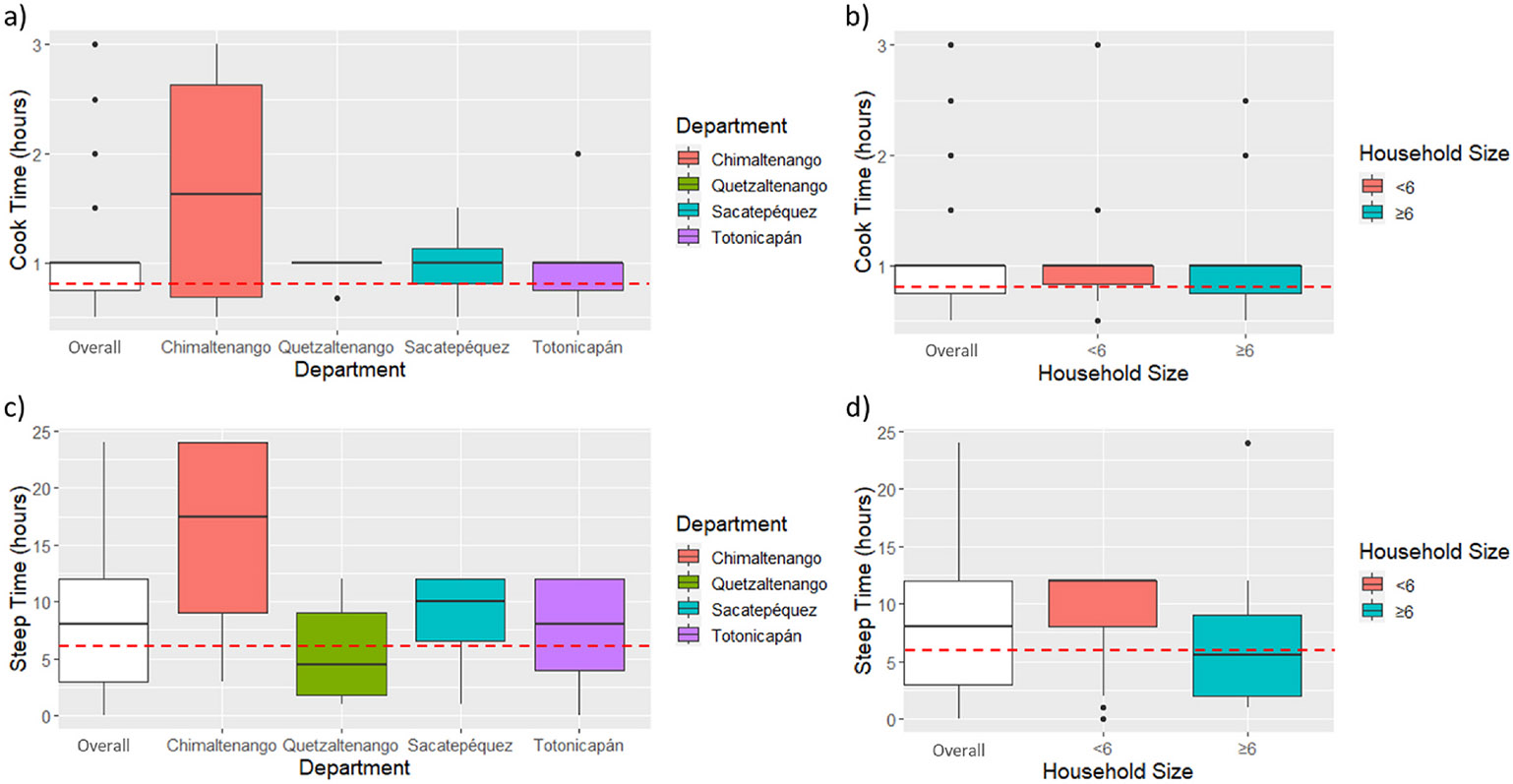
A–d. Cook and steep times by department and household size. Dashed lines denote cook time suggested minimum (40 min) and steep time suggested minimum (6 h).

When preparing the maize for nixtamalization, no households crack the maize kernels prior to cooking and only two households (6 %) would be open to trying. For adding the cal (calcium hydroxide) to the maize, the majority of households (91 %) combine the cal and water first while 30 % boil the water and cal mixture prior to adding to the maize. Every household reported using a wood stove and allowing the nixtamal to boil while 78.8 % covered the cooking nixtamal with a lid. Fig. 2 and S1-S8 depict box plots of the different proportions of water, cal, and maize, cooking time, and steeping times overall and broken down by demographics. Interestingly, for household size, those households that were larger (≥6 household members) had lower steep times while also producing smaller batches of tortillas or tamales. For departments, cook and steep times were similar except for households in Chimaltenango which had above average times while having below average batch sizes of tortillas or tamales (see Fig. 2a and c). Finally, there was less variation in cook and steep times as well as batch sizes across households that spoke different languages.

Tap water was the most common source of water used for cooking (78.8 %), with well water (18 %) and tank water (3 %, n = 1) also reported. The median number of times households wash the nixtamal after steeping was three (range 0–7). The number of washes ranged from a median of two among households in Sacatepéquez to a median of four among households in Totonicapán. Most households did not recycle the cooking water (nejayote) or wash water for any other uses (84.8 %), while watering plants (n = 4) or animals (n = 2) were reported. Every household reported using a community mill to grind their nixtamalized maize into masa.

**Fig. 3.**
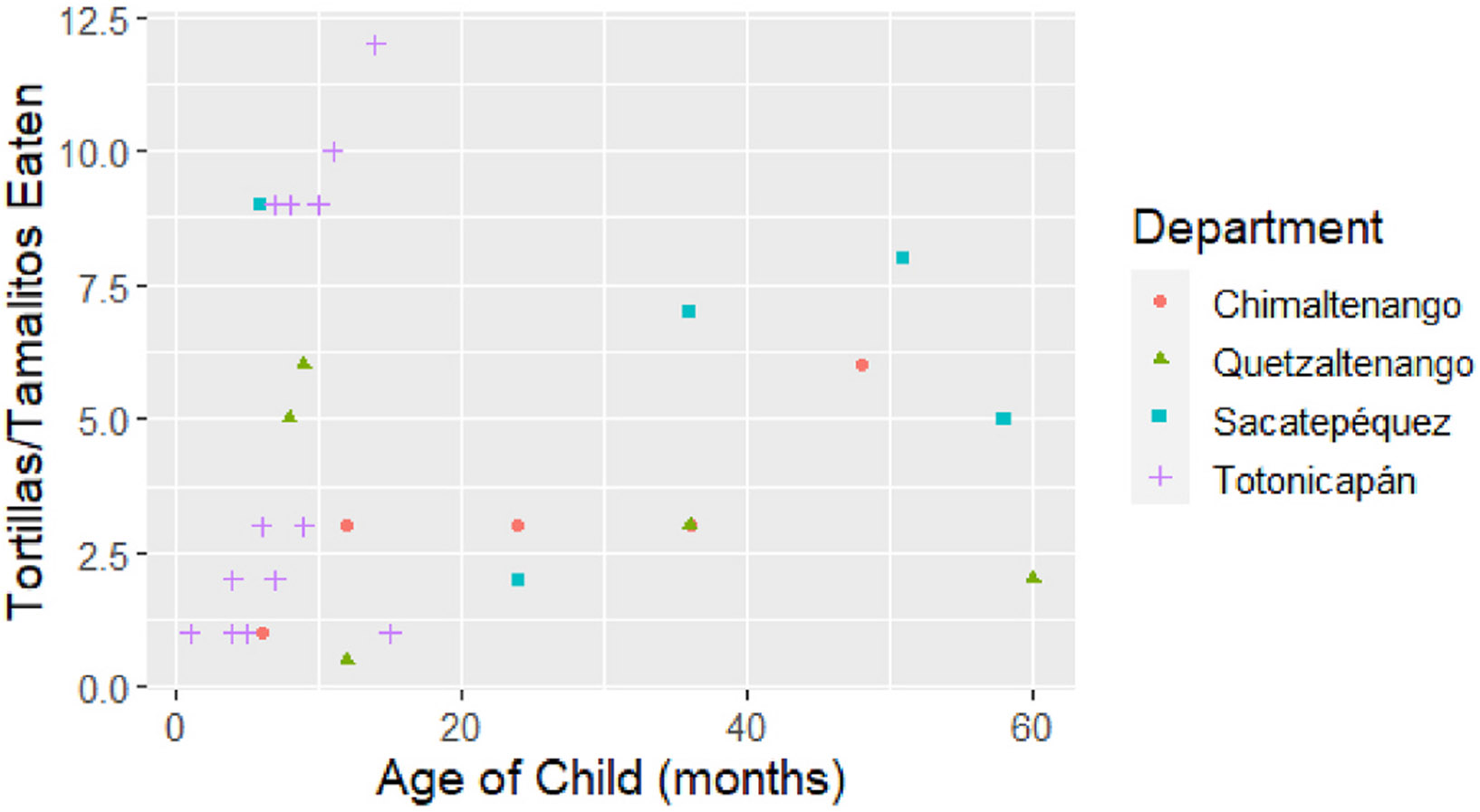
Tortilla or tamalito consumption per day by children (0–60 months) among households.

Finally, the mean number of tortillas or tamales consumed per household member per day was 6.6 (median 5.7; range: 0.6–16). Fig. 3 and S9-S10 depict scatter plots of tortilla or tamale consumption per day by children in the households. Generally, as children age, tortilla or tamale consumption also increases, but there is significant variation. Interestingly, children in households from Totonicapán consume a lot of tortillas or tamales early on while the number of tortillas or tamales consumed by children in Quetzaltenango remains low as children get older. Finally, households were also asked about other maize-based foods they prepare, these are listed in Table 2. The majority of these products are made using maize that has been nixtamalized except for ‘pinol’, which is often made from ground, roasted maize-kernels.

## 4. Discussion

### 4.1. Maize post-harvest practices and storage

Smallholder practices around maize acquisition (cultivation and purchasing), post-harvest, and storage are critical for controlling fungal growth that can lead to fungal toxin exposure. Garsow and colleagues reviewed the recent literature around pre- and post-harvest maize practices associated with mycotoxin contamination in the Guatemalan context [9]. In the Western Highlands, harvest typically occurs between November and December; this is supported by our data with a few earlier and later outliers. Drying the maize post-harvest is critical for fungal control. The US Department of Agriculture Extension offices recommends a maximum moisture content of maize of 13–15 % depending on planned storage time [13,14]. As monitoring devices are limited in settings similar to our survey location, maximizing drying is critical. Previous literature from Huehuetenango reported that 93.5 % of households dry their maize before storage [15]. While our survey did not include this specific metric, 97 % of households did report that they were intentional about keeping their maize dry, providing examples including drying in the sun and finding dry places to store the maize. This suggests there is demand by households for methods to ensure maize is dried and stored appropriately. However, effectiveness in storage or in reducing moisture content of the maize for each of the drying methods used by households was not evaluated in any of the studies.

We also collected data on what proportion of households stored their maize still on the cob compared to shelling (or shucking) the maize to store just the kernels. Each approach has implications on best practices for drying and storing the maize. Our data suggest 64 % of household shell or shuck all of their maize while 36 % retain at least some portion of the harvest in cob form. Furthermore, some households have reported leaving the husks on the cob of un-shelled maize [15]. Ensuring clear and data-driven guidance is available for all approaches given the wide range of drying and storage resources available to households is critical. Maize left on the cob can help mitigate the spread of fungi if one cob is infected, but complete drying may be more difficult and require slightly longer drying times as compared to shelled maize. For shelled maize, rotten kernels should be screened and removed to ensure spread of fungi does not occur. Finally, previous data suggest that the most common storage systems used in Guatemala include sacks, wooden boxes (“*trojas*”), hanging from the ceiling (“*tapanco*”), or silos [16,17]. These were also commonly reported in our study but also included barrels (primarily plastic). It is important that regardless of the storage system, maize remain dry, humidity is kept low, and, where possible the storage system promote proper aeration/ventilation to enable uniform temperatures throughout the system [13]. Temperature gradients can cause moisture pockets and buildup, resulting in hospitable regions for fungal growth. Interestingly, of those households that both cultivated and bought maize at some point during the year, 89 % of households reported storing the maize in the same location. This can be an important factor in cross-contamination if one source of maize is at higher risk of fungal contamination [18].

Our results and established guidelines can be helpful in developing resources and communication materials for agricultural extension programming in Guatemala. The primary metrics reported here appeared consistent across geography suggesting having a diverse range of material to support households with is important. Households in Totonicapán did appear to have a higher rate of not shelling their maize, but additional data should be collected to verify this trend across a larger sample of the population. Understanding household maize handling practices helps to support households in optimizing practices to maximize control of fungal contamination and reduce toxin exposure.

### 4.2. Nixtamalization processes

Nixtamalization practices are less well studied in Guatemala, however, empirical data from laboratory studies of nixtamalization can provide insight into recommended ranges for parameters to maximize reduction of aflatoxin in the resulting maize-based food product. Schaarschmidt and Fauhl-Hassek provide a review of nixtamalization on the reduction of aflatoxin in maize [10] and we provided a general set of thresholds for different steps in the nixtamalization process based on this review. Across the reviewed studies, there is a wide range of reported effectiveness, given the diversity of contexts, maize and fungus strains, and study designs. However, the previously reported data suggests that variations in the concentrations of cal used in the process may have a minimal effect of the reduction levels of aflatoxin as long as a minimum threshold is achieved (we suggest at least 0.33 ratio cal-to-water by mass). However, anecdotal data from our project suggested households typically use visual indicators to identify the correct amount of cal to add. Translating numerical threshold values into visual indicators, while difficult, could benefit households.

For cooking and steeping times, studies reviewed by Schaarschmidt and Fauhl-Hassek 2019 suggested that a minimum of 40 min of cooking and a minimum of 6 h of steeping are important for obtaining AFB1 reductions above 70 %, but longer times generate better reductions. In our survey, the mean nixtamal cooking time was 1 h with n = 4 households below the recommended minimum of 40 min. The median steeping time was 8 h with n = 8 households reporting <6 h. For those households with more members, cooking and steeping times were lower. Further research on Guatemalan-specific nixtamalization practices would help stakeholders in developing support materials for households to optimize their practices for maximum fungal toxin reduction given local feasibility and cultural context.

In our study, the primary nixtamalized food-products were either tortillas (primarily in Chimaltenango and Sacatepéquez) or tamales (primarily in Quetzaltenango and Totonicapán). For preparing the maize, cracking the hard maize kernels is hypothesized to improve the effectiveness of the cal solution (calcium hydroxide) penetrating the kernel and reducing the aflatoxin, however, no households practiced this and very few households were interested in trying.

Households washed their maize a mean of three times. Previous literature suggests that washing is important for helping remove aflatoxin from the nixtamal, however, washing also lowers the pH of the nixtamal which is a primary mechanism of detoxification of aflatoxin [10]. In our survey, only one household did not wash their nixtamal (only drained the nejoyte) while n = 7 households washed their maize greater than 4 times. The majority of households also reported immediately disposing of the nejoyte (cooking water) and wash water after use, which is recommended as previous data has shown that if the aflatoxin is present in the maize, it will be partially removed via this water. However, several households utilized it for watering plants or animals. Finally, all households reported using a community mill to grind the nixtamal into masa, however, no data exist on the quality and hygiene practices of these mills. Further data on the risk of cross-contamination of fungal toxins or other contaminants between batches of maize could help identify cost-effective best practices for these community mills.

## 5. Conclusion

This pilot study provides household-level insight to both maize handling and traditional nixtamalization practices utilized by local households across geographical and cultural settings in Guatemala. However, this study was limited on sample size and geographic region given available resources and we encourage further work especially in understanding local nixtamalization practices.

The findings highlight crucial insights for field workers and agricultural extension agents aiming to enhance maize handling and nixtamalization practices in rural Guatemala. First, the variability in post-harvest and storage practices suggests a need for localized, culturally sensitive interventions that consider seasonal and geographic differences. Implementing tailored educational programs that emphasize the benefits of proper drying and storage techniques can mitigate aflatoxin contamination risks. Second, promoting optimized nixtamalization processes – such as sufficient cooking and steeping times – can improve exposure mitigation. While the majority of households reported practicing nixtamalization steps at or above previously reported thresholds, identifying sustainable and economic approaches for all households to use these practices is critical. Furthermore, extension services should prioritize knowledge dissemination on safe water usage and the disposal of nejayote to prevent aflatoxin exposure. Addressing these gaps can empower households to adopt safer maize processing methods and inform the development of policies and programs that safeguard public health while respecting cultural practices.

## Supplementary Material

1

2

## Figures and Tables

**Table 1:** Descriptive characteristics of Household’s maize handling, storage, and nixtamalization practices.

Variable	Prevalence
Department	Chimaltenango: 4
	Quetzaltenango: 8
	Sacatepéquez: 8
	Totonicapán: 13
Language	Kaqchikel: 4
	Mam: 3
	K’iche’: 15
	Spanish: 11
Household members	Median: 6 (range: 1–11)
	<6: 16
	≥6: 17
Most common harvest month	November
Consumed only cultivated maize in past 30 days	11 (33 %)
Consumed a combination cultivated and purchased maize in past 30 days	21 (64 %)^a^
Mean nixtamal cook time (hr)	1 (range: 0.5–3)
Mean nixtamal steep time (hr)	8 (range: 1–24)

Maize sources, handling, and storage.

a One household consumed only purchased maize in the past 30 days.

**Table 2:** List of other maize-based products households make in Guatemala.

Maize-Based Food	Description
Atole	A traditional hot beverage made from masa (corn dough), water, and occasionally flavored with cinnamon, chocolate, or fruit.
Agua de tortilla	A traditional drink made by blending toasted tortillas with water, it’s often flavored with salt or sugar.
Agua de masa	Water mixed with dissolved corn dough, used as a base for soups or drinks, sometimes seasoned or sweetened.
Tamales con recado or chuchitos	Tamales are steamed corn dough wrapped in corn husks, with ‘recado’ referring to a savory sauce filling. Chuchitos are a simpler, smaller version, often with a tomato-based sauce and meat.
Tamalitos de chipilin or tayuyos	Small tamales flavored with chipilin, an herb, or filled with a mix of ingredients (like beans) wrapped in corn dough.
Maize frito (tostadas, dobladas)	Fried maize dishes; tostadas are flat, crispy tortillas topped with various ingredients, while dobladitas are folded and stuffed.
Empanadas	Stuffed pastries made from masa and typically filled with meat, cheese, or other ingredients, then fried or baked.
Pinol	A dry powder made from toasted maize kernels, often mixed with water to make a beverage or used as a culinary ingredient.

## Data Availability

Data will be made available on request.
