# Supplementary material for: Practices of maize handling and nixtamalization to reduce fungal toxin exposure in rural Guatemala
Source: J Agric Food Res. Author manuscript; Available in PMC 2025 Jan 10. (PMC11722548; doi:10.1016/j.jafr.2024.101512)

**Supplementary Material**

“*Practices of Maize Handling and Nixtamalization to Reduce Fungal Toxin Exposure in Rural Guatemala*”

**Table S1. Descriptive Statistics of Survey Data with Breakdowns by Demographics**

| **Variable** | **Overall** | **Department** | | | | **Language** | | | **Household Members** | |
| --- | --- | --- | --- | --- | --- | --- | --- | --- | --- | --- |
|  | **(n=33)** | **Chim. (n=4)** | **Quetz. (n=8)** | **Sacat. (n=8)** | **Toto. (n=13)** | **Span. (n=11)** | **Quiché (n=15)** | **Other (n=7)** | **<6**  **(n=16)** | **≥6**  **(n=17)** |
|  |  |  |  |  |  |  |  |  |  |  |
| Amount of corn (kgs) | Mean 3.19 (2.16)  Median 2.27 (0.454-9.07) | Mean 1.59 (0.868)  Median 1.81 (0.454-2.27) | Mean 3.03 (1.09)  Median 3.31 (1.81-4.54) | Mean 2.04 (1.06)  Median 2.04 (0454-3.63) | Mean 4.49 (2.71)  Median 4.08 (0.907-9.07) | Mean 2.23 (1.17)  Median 1.81 (0.454-4.54) | Mean 4.26 (2.61)  Median 3.63 (0.907-9.07) | Mean 2.43 (1.25)  Median 2.27 (0.454-4.00) | Mean 2.49 (1.99)  Median 2.18 (0.454-9.07) | Mean 3.19 (2.16)  Median 2.27 (1.36-9.07) |
| Color of Corn | 17 Yellow  16 White | 3 Yellow  1 White | 5 Yellow  3 White | 1 Yellow  7 White | 8 Yellow  5 White | 3 Yellow  8 White | 9 Yellow  6 White | 5 Yellow  2 White | 10 Yellow  7 White | 7 Yellow  9 White |
| Is the maize cracked before cooking? (Yes, No) | 33 No  0 Yes | 4 No  0 Yes | 8 No  0 Yes | 8 No  0 Yes | 13 No  0 Yes | 11 No  0 Yes | 15 No  0 Yes | 7 No  0 Yes | 16 No  0 Yes | 17 No  0 Yes |
| Willing to crack maize? (Yes, No, Don’t know) | 2 Don’t know  29 No  2 Yes | 0 Don’t know  4 No  0 Yes | 0 Don’t know  8 No  0 Yes | 1 Don’t know  5 No  2 Yes | 1 Don’t know  12 No  0 Yes | 1 Don’t know  9 No  1 Yes | 1 Don’t know  14 No  0 Yes | 0 Don’t know  6 No  1 Yes | 2 Don’t know  14 No  1 Yes | 0 Don’t know  15 No  1 Yes |
| Amount of water (liters) | Mean 6.34 (4.42)  Median 5.00 (2.00-18.9) | Mean 6.00 (2.71)  Median 5.00 (4.00-10.0) | Mean 8.51 (5.88)  Median 7.00 (3.00-18.9) | Mean 4.95 (2.71)  Median 4.00 (2.00-10.0) | Mean 5.97 (4.61)  Median 5.00 (2.00-18.9) | Mean 5.60 (3.27)  Median 4.00 (2.00-10.0) | Mean 6.18 (4.24)  Median 5.00 (2.00-18.9) | Mean 7.87 (6.39)  Median 5.00 (3.00-18.9) | Mean 5.50 (4.63)  Median 4.00 (2.00-18.9) | Mean 7.24 (4.14)  Median 5.50 (2.00-18.9) |

**Figure S1**. Cook and Steep Time by Primary Language Spoken


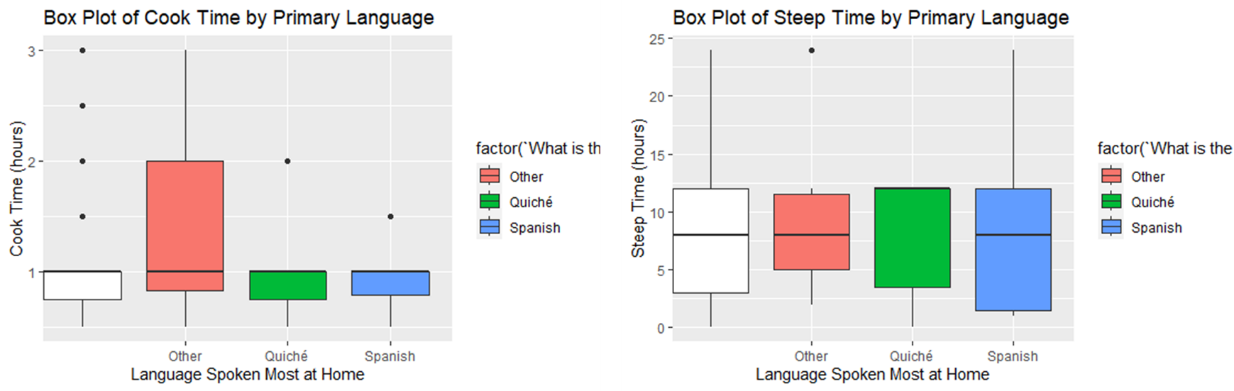


**Figure S2**. Tortillas and Tamalitos Produced Per Batch by Household Size


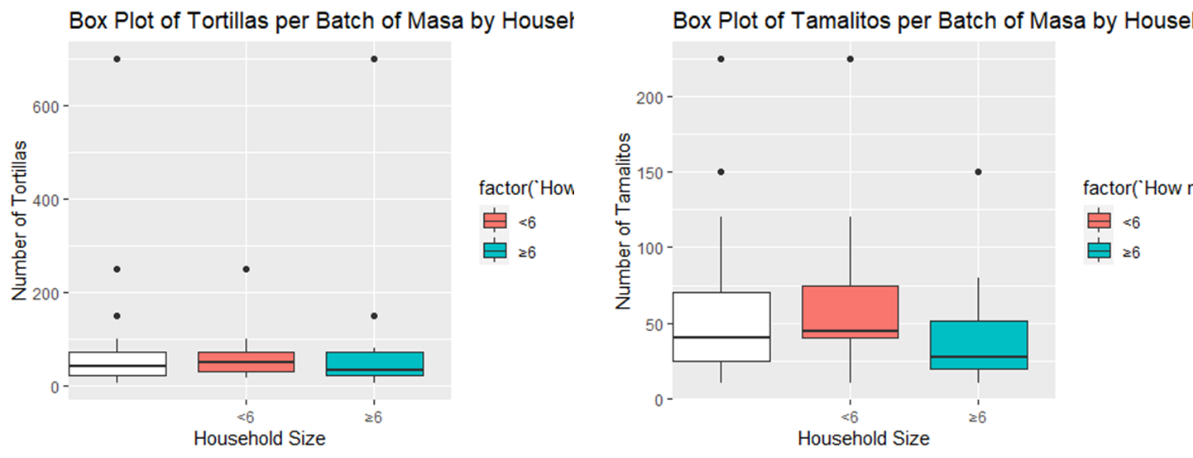


**Figure S3**. Tortillas and Tamalitos Produced Per Batch by Department and Language Spoken


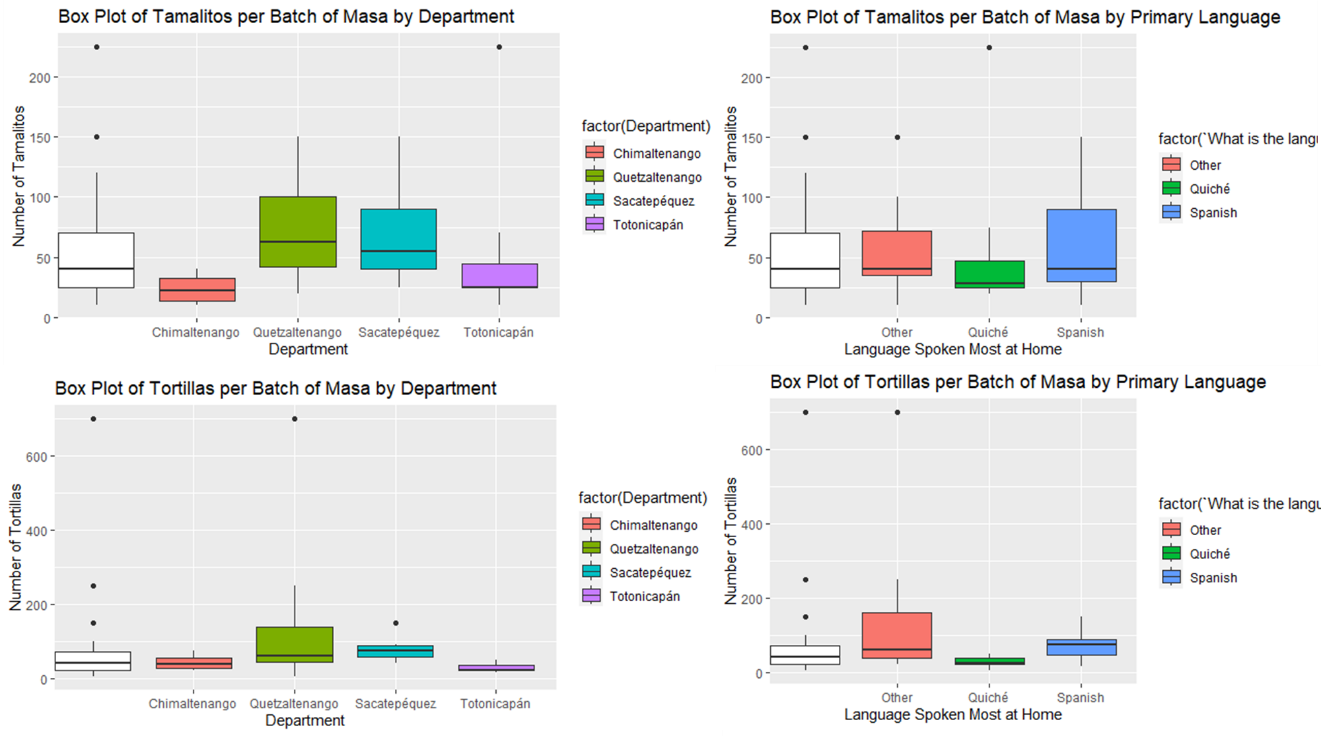


**Figure S4**. Amount of Maize and Water Used by Household Size


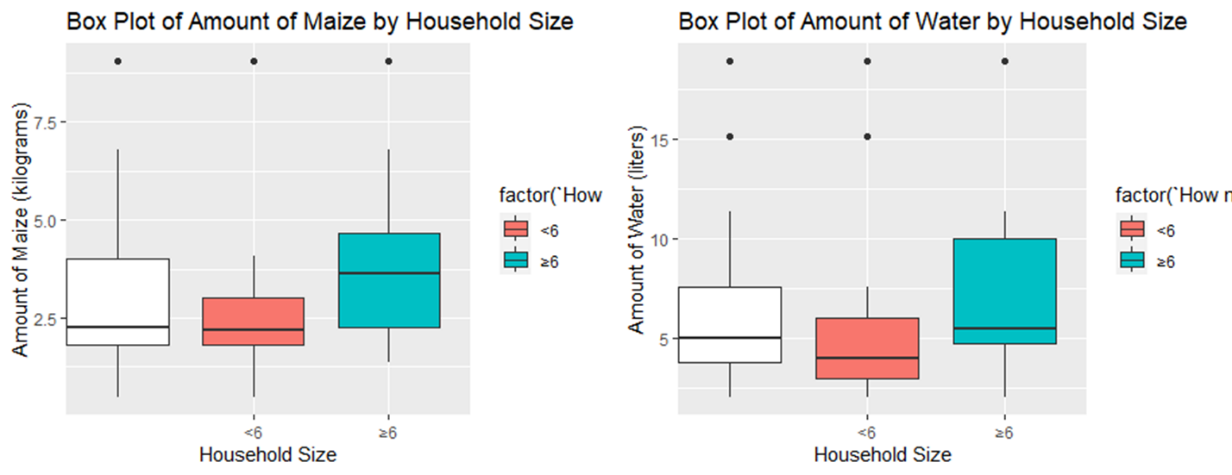


**Figure S5**. Amount of Maize and Water Used by Department and Language Spoken


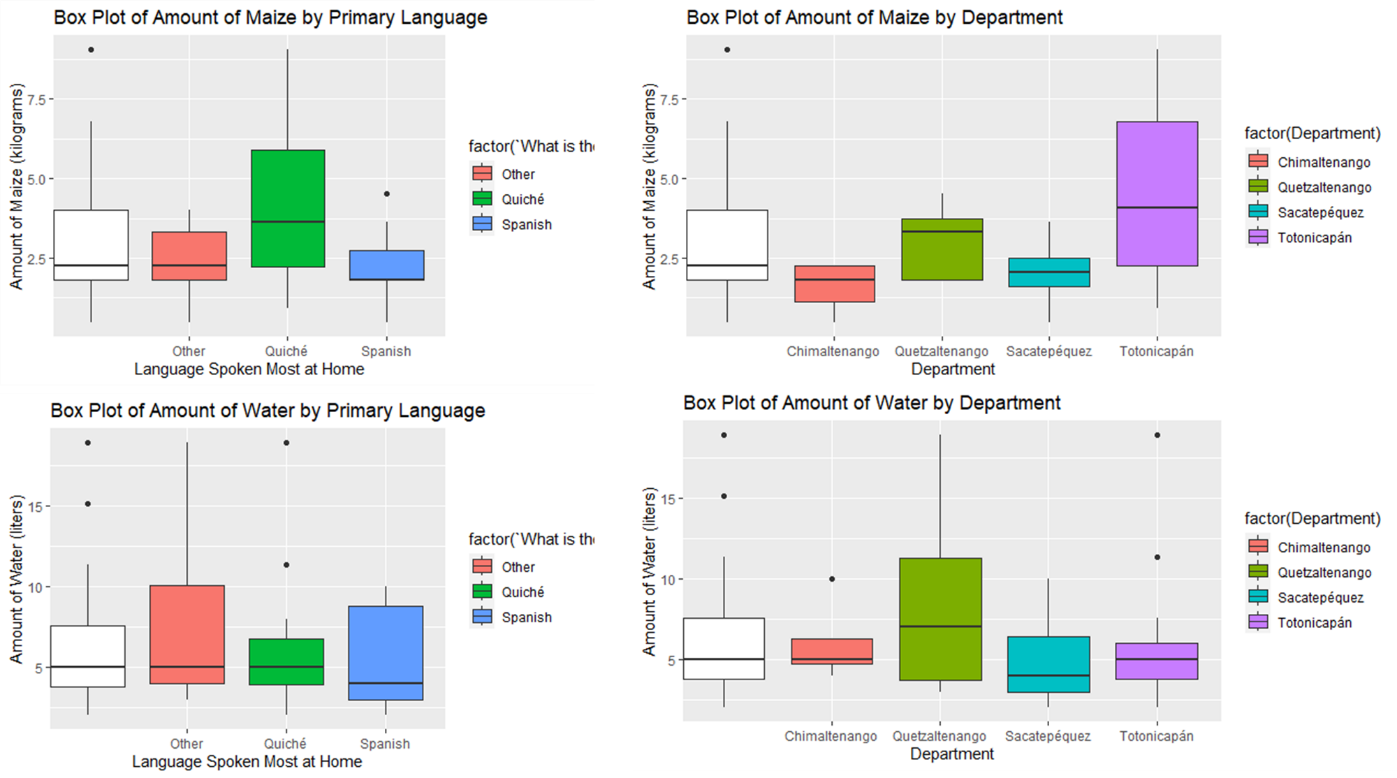


**Figure S5**. Cal-Water-Maize Ratios by Household Size

**Figure S6**. Cal-Water-Maize Ratios by Department and Language Spoken

**Figure S7**. Number of Times Nixtamal is Washed by Household Size

**Figure S8**. Number of Times Nixtamal is Washed by Department and Language Spoken

**Figure S9**. Tortilla or Tamalito Consumption Per Day by Children among Households by Household Size


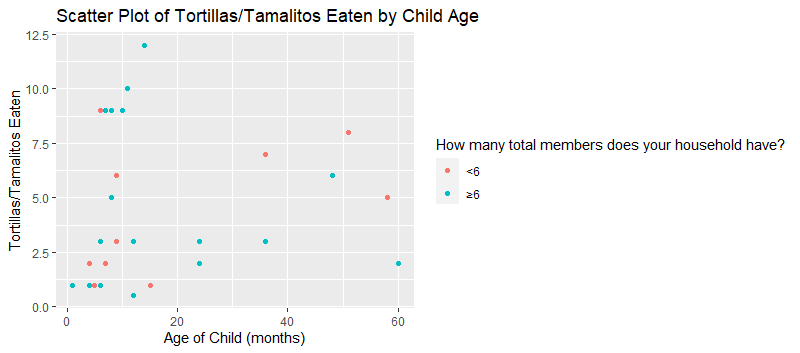


**Figure S10**. Tortilla or Tamalito Consumption Per Day by Children among Households by Language Spoken


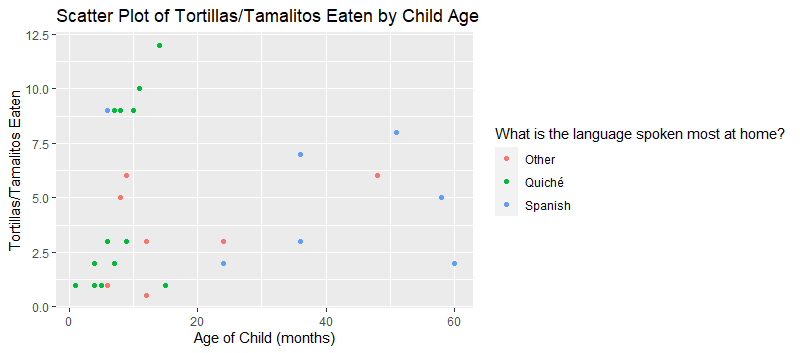

Supplement: 1 [file NIHMS2042453-supplement-1.docx]
