# Supplementary material for: Practices of maize handling and nixtamalization to reduce fungal toxin exposure in rural Guatemala
Source: J Agric Food Res. Author manuscript; Available in PMC 2025 Jan 10. (PMC11722548; doi:10.1016/j.jafr.2024.101512)
Supplement: 2 [file NIHMS2042453-supplement-2.docx]

**Supplemental Material: Spanish Version of Manuscript**

**Prácticas de manejo del maíz y nixtamalización para reducir la exposición a toxinas fúngicas en zonas rurales de Guatemala**

Gabriela Montenegro-Benthancourt^1^, Hannah Glesener^2,3^, Olga Torres^4,5^, Emeline Seegmiller^2^, Peter Rohloff^1^, Lee E. Voth-Gaeddert^1,2^

^1^ Center for Indigenous Health Research, Wuqu’ Kawoq|Maya Health Alliance, Tecpan, Chimaltenango 04006, Guatemala

^2^ Biodesign Center for Health Through Microbiomes, Arizona State University, Tempe, AZ 85281, USA

^3^ School for Engineering of Matter, Transport, and Energy, Arizona State University, Tempe, AZ 85281, USA

^4^ Laboratorio Diagnóstico Molecular, Guatemala City, 01015, Guatemala

^5^ Centro De Investigación en Nutrición y Salud, Guatemala City, 01015, Guatemala

**Resumen**

Las toxinas fúngicas en los suministros alimentarios locales son un riesgo ambiental crítico para la salud de las comunidades a nivel mundial. Para mejorar la caracterización de los puntos de control de toxinas hipotéticos entre los hogares, realizamos encuestas de hogares en cuatro departamentos (primera división administrativa) en Guatemala. Los datos recopilados incluyeron la cosecha, el procesamiento, el almacenamiento y las prácticas tradicionales de nixtamalización del maíz. En total, participaron en la encuesta n=33 hogares de 4 departamentos únicos, 17 municipios únicos y representaban 4 idiomas diferentes. Los resultados sugirieron que la mayoría de los hogares consumían una combinación de maíz cultivado personalmente y comprado. Hubo una variación significativa en la forma en que se almacenó este maíz, en lo que respecta al preprocesamiento (grano vs mazorca entera), así como en el tipo de sistema de almacenamiento. Para la nixtamalización, las mayores diferencias en las prácticas (por ejemplo, tiempo de cocción) se basaron en el tamaño del hogar, mientras que la mayoría de los hogares reportaron prácticas que se alineaban con las prácticas mejoradas reportadas anteriormente. Por último, todos los productos alimenticios a base de maíz producidos por hogares reportaron que utilizaban el proceso de nixtamalización excepto uno. Las prácticas actuales de manejo del maíz y nixtamalización reportadas por la mayoría de los hogares coinciden con las prácticas mejoradas; sin embargo, con una guía adaptada a las necesidades locales y culturalmente sensible difundida por las partes interesadas, se puede mejorar la prevalencia del uso de las prácticas mejoradas entre los hogares. Una mayor investigación comunitaria sobre las prácticas tradicionales de cultivo y nixtamalización puede mejorar estas recomendaciones.

**Palabras clave:** aflatoxina; seguridad alimentaria; salud pública; almacenamiento de maíz; tortillas

**Introducción**

La seguridad alimentaria de los cultivos básicos es un aspecto fundamental para la salud, el crecimiento y el desarrollo de los niños (1). En Guatemala, como en muchos países de América Latina, el maíz es un cultivo importante tanto desde una perspectiva de agricultura industrial como de subsistencia, y representa el 70% de la dieta de algunas comunidades (2). Las toxinas fúngicas, como la aflatoxina, pueden contribuir a pérdidas económicas significativas en las operaciones de agricultura industrial y de subsistencia y plantean un riesgo significativo para la salud a través del consumo humano de maíz (3,4). La aflatoxina B1 (AFB1) es un carcinógeno de clase 1 que causa cáncer de hígado con la exposición a largo plazo, mientras que los efectos de la exposición a corto plazo son menos conocidos (5). Identificar y fortalecer los puntos críticos de control y los enfoques para reducir la transmisión de toxinas fúngicas en las cadenas de valor regionales y nacionales del maíz es importante pero complicado debido a las numerosas partes interesadas y sus prioridades y obligaciones en conflicto (6-8). Si bien el progreso para fortalecer las cadenas de valor del maíz continúa a nivel regional y nacional, también se puede empoderar aún más a los hogares para que mejoren la calidad de su maíz de subsistencia y sus prácticas de cocción para reducir las posibles exposiciones a toxinas fúngicas peligrosas.

Desde la perspectiva de los hogares, estos tienen a su disposición los siguientes puntos de control para mitigar el crecimiento y la exposición a toxinas fúngicas: salud de los cultivos antes de la cosecha, prácticas de procesamiento después de la cosecha, prácticas de almacenamiento después de la cosecha, selección de maíz en el mercado, prácticas de almacenamiento de maíz comprado y cocción del maíz (es decir, nixtamalización) (7). Existen amplios recursos que brindan orientación sobre las prácticas mejoradas en los aspectos agrícolas de estos puntos de control. Garsow y sus colegas brindan una descripción general para Guatemala de estos puntos de control y enfoques de control de "prácticas mejoradas" para el cultivo, la cosecha y el almacenamiento del maíz (9). Hay menos recursos disponibles para brindar orientación sobre las prácticas mejoradas para la nixtamalización. Schaarschmidt y Fauhl-Hassek brindan una revisión de los parámetros óptimos para la nixtamalización para reducir la biodisponibilidad de toxinas fúngicas con base en estudios previos (10). Si bien destacan una variación significativa en estos estudios, se pueden extraer umbrales generales. Para la AFB1, proponemos los siguientes umbrales:

- Cantidad de hidróxido de calcio (cal): una relación de cal a agua de al menos ~0,33 (p. ej., 1 parte de cal por 300 partes de agua; en masa)
- Tiempo de cocción del maíz: >40 minutos; especialmente si el tiempo de remojo es corto (<12 horas)
- Tiempo de remojo del maíz: >6 horas, pero cuanto más tiempo, mejor, 8 horas o más (1 hora reduce la AFB1 en ~50%; mientras que 12 horas la reduce en ~90%)
- Lavado del maíz cocido (nixtamal): ≥1 vez

Si bien es útil, se recomienda una mayor confirmación de estos umbrales. Además, para traducir estos umbrales y optimizar las estrategias de programación y participación de los agentes de extensión de salud comunitaria o agrícola, es importante comparar la variación dentro de las prácticas domésticas actuales con las prácticas mejoradas propuestas.

En primer lugar, los puntos de control de hongos y toxinas dentro de las cadenas de valor locales del maíz están disponibles para los hogares, pero a menudo cambian debido a factores estacionales (momento de la cosecha, precio de mercado del maíz, cambio de fuente de maíz, capacidad de almacenamiento y calidad, etc.). Si bien la programación nacional uniforme puede hacer que la implementación del programa sea más sencilla y económica, la creación de programas más adaptados geográficamente puede ser más rentable debido a una mejor alineación con las necesidades de los hogares y las barreras de implementación. Por lo tanto, es importante comprender la variación en las prácticas agrícolas actuales en las distintas geografías y culturas. En segundo lugar, los procesos de nixtamalización son una práctica cultural importante, así como un proceso eficaz para la desintoxicación de toxinas fúngicas peligrosas (aflatoxina B1 y fumonisina B1) (10). Lamentablemente, estas prácticas han recibido poca atención de los esfuerzos de programación regional o nacional, posiblemente debido a las dificultades para 1) definir las mejores prácticas y 2) comprender las prácticas actuales y las barreras al cambio entre los hogares en distintas geografías, culturas y niveles económicos.

En este artículo, informamos sobre los resultados de una encuesta de hogares realizada por agentes de extensión agrícola locales en cinco departamentos del oeste de Guatemala para identificar las prácticas actuales de los hogares en materia de cosecha, procesamiento y almacenamiento de maíz, así como los procesos de nixtamalización del maíz. El objetivo de esta encuesta es 1) proporcionar información crítica a las partes interesadas en la implementación sobre las prácticas actuales y 2) demostrar un enfoque de bajo costo para llenar los vacíos de datos para mejorar la programación regional y nacional con el fin de mejorar la salud de la población.

**Materiales y métodos**

*Sitio del estudio*

Guatemala es el país más grande de América Central y alberga una población diversa de más de 20 etnias mayas diferentes, así como ladinos, xincas y otras etnias no mayas. El maíz es el cultivo básico y el principal cultivo de los agricultores de subsistencia. El altiplano occidental de Guatemala es montañoso con dos estaciones principales (lluviosa y seca) que resultan en una temporada de cosecha por año, mientras que en las tierras bajas y las regiones costeras existen dos temporadas de cosecha (11). Esta dinámica a menudo influye en los fluctuaciones estacionales en la cadena de suministro de maíz, con grandes porciones de maíz que provienen de las regiones costeras o México durante los períodos de escasez del año en el altiplano occidental. Las comunidades rurales montañosas a menudo son económicamente más pobres y tienen tasas más altas de desnutrición crónica entre los niños menores de cinco años (12). Dada la variación de las prácticas culturales y las geografías, Guatemala proporciona un lugar importante para demostrar métodos para mejorar la programación de apoyo a los hogares a nivel regional y nacional.

**Figura 1.** Mapa de Guatemala y ubicación de los hogares encuestados.
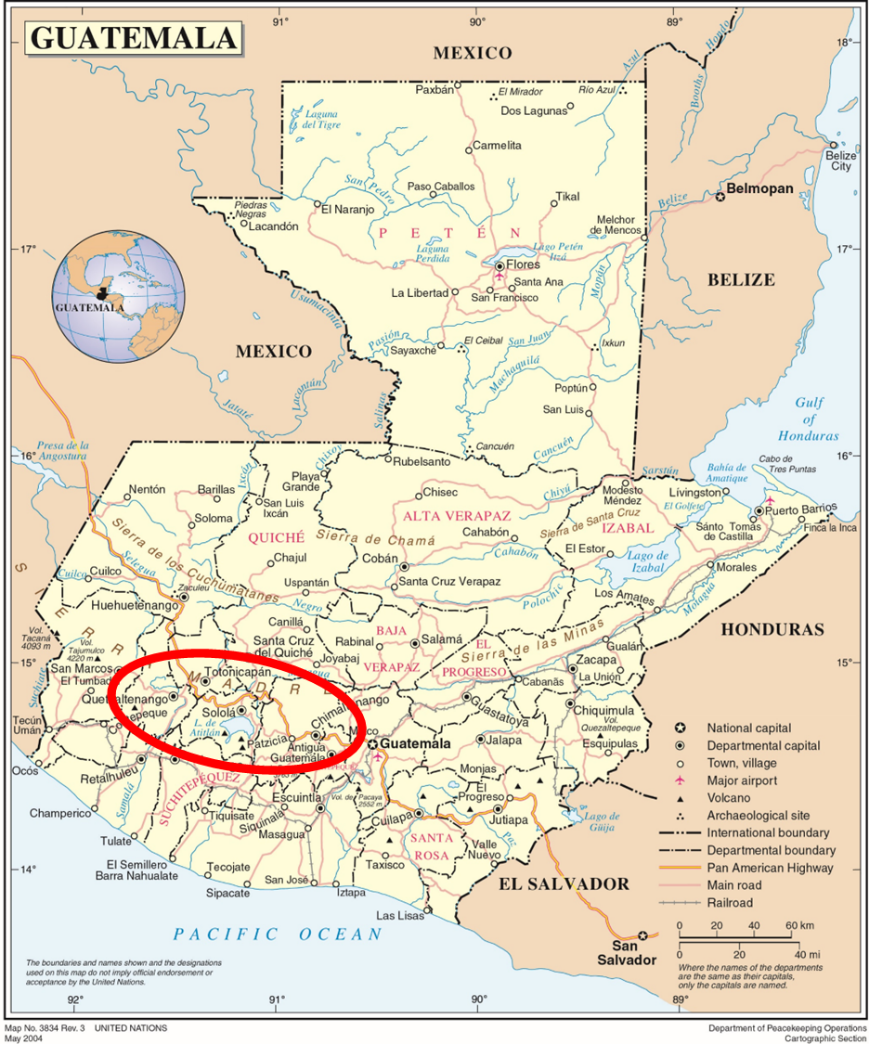


*Gráfico adaptado de los archivos geoespaciales de las Naciones Unidas.*

*Encuesta*

En julio y agosto de 2023, los agentes de extensión agrícola del Ministerio de Agricultura, con el apoyo del Cuerpo de Paz de los Estados Unidos y la Maya Health Alliance, realizaron una encuesta de hogares para recopilar información sobre las prácticas actuales de poscosecha, almacenamiento y procesos de nixtamalización del maíz (ver Figura 1). El equipo de investigación construyó la encuesta en español e inicialmente se puso a prueba entre un pequeño grupo de hogares. Luego, los agentes de extensión administraron la encuesta final de forma oral a un miembro del hogar y los datos se registraron en formularios de encuestas en papel. Los datos se transfirieron a formato digital para su análisis posterior. Las comunidades se seleccionaron con base en un gradiente geográfico con el objetivo de incorporar cuatro departamentos y al menos tres municipios únicos dentro de cada departamento. Los hogares se seleccionaron con base en una muestra de conveniencia dentro de la comunidad. Esta estrategia de muestreo se utilizó para maximizar la variación potencial observada entre los hogares en las prácticas de manejo del maíz y los procesos de nixtamalización. El instrumento de la encuesta se puede encontrar en el material complementario tanto en español como en inglés. El estudio recibió una determinación de exención (investigación con sujetos no humanos) de Wuqu' Kawoq | Junta de Revisión Institucional de Maya Health Alliance (WK-2023-002).

*Análisis de datos*

Se generaron estadísticas descriptivas para todos los datos recopilados, incluidas la media y la desviación estándar, la mediana y el rango, y los diagramas de caja, según corresponda. Además, las variables recopiladas se estratificaron según la geografía, el idioma principal hablado y la cantidad de miembros del hogar para identificar visualmente la posible variación impulsada por la ubicación, la etnia o las situaciones económicas/de vida. Se utilizaron pruebas estadísticas bivariadas adecuadas para evaluar las diferencias significativas entre los grupos (por ejemplo, la geografía) de las variables recopiladas. Los datos se analizaron utilizando Excel v2401 y R v4.3.1.

**Resultados**

Entre julio y agosto de 2023, se encuestaron n=33 hogares. Los hogares representaban cuatro departamentos diferentes y 17 municipios diferentes. Se informó que cuatro idiomas diferentes eran el idioma principal hablado en los hogares (español, k’iche’, kaqchikel y mam) y la mediana del número de miembros que vivían en los hogares era seis (rango: 1-11). La Tabla 1 y la Tabla S1 presentan estadísticas descriptivas para las variables recopiladas en general y desglosadas por estos grupos demográficos.

**Tabla 1.** Características descriptivas de las prácticas de manipulación, almacenamiento y nixtamalización del maíz en los hogares

| **Variable** | **Predominio** |
| --- | --- |
| Departamento | Chimaltenango: 4  Quetzaltenango: 8  Sacatepéquez: 8  Totonicapán: 13 |
| Idioma | Kaqchikel: 4  Mam: 3  K’iche’: 15  Spanish: 11 |
| Miembros del hogar | Mediana: 6 (Rango: 1-11)  <6: 16  ≥6: 17 |
| Mes de cosecha más común | Noviembre |
| Consumió solo maíz cultivado en los últimos 30 días | 11 (33%) |
| Consumió una combinación de maíz cultivado y comprado en los últimos 30 días | 21 (64%)* |
| Tiempo medio de cocción del nixtamal (horas) | 1 (rango: 0.5-3) |
| Tiempo medio de remojo del nixtamal (horas) | 8 (rango: 1-24) |

**Un hogar consumió únicamente maíz comprado en los últimos 30 días.*

*Fuentes, manejo y almacenamiento de maíz*

En cuanto a las fuentes de maíz utilizadas en los hogares en los últimos 30 días, el 70% (23/33) de los hogares informó haber consumido maíz cultivado como su fuente principal, mientras que el 97% de los hogares practicaba el cultivo de maíz en algún grado. Sin embargo, solo el 33% de los hogares informó haber consumido únicamente maíz cultivado por ellos mismos, mientras que el 67% de los hogares consumía una combinación de maíz cultivado y maíz de mercado o solo maíz de mercado. Solo el 12% de los hogares informó haber consumido maíz en los últimos 30 días solo proveniente del mercado. El mes de cosecha más común en el año anterior fue noviembre, pero varió de septiembre a febrero. Los hogares en Chimaltenango informaron fechas de cosecha más tardías (diciembre-febrero) en comparación con los otros tres departamentos.

Para el procesamiento posterior a la cosecha, el 64% de los hogares desgrana todo su maíz, mientras que el 21% de los hogares solo desgrana el maíz que consumirá inmediatamente (el 12% se encuentra en algún punto intermedio; 1 hogar solo compra maíz). De los n=11 hogares que informaron dejar parte o todo el maíz en la mazorca para almacenar, 8 de 11 de esos hogares eran de Totonicapán. Si los hogares desgranan todo su maíz (n=20), las ubicaciones de almacenamiento reportadas incluyeron silos (8/20), sacos (6/20), barriles o cajas (5/20) o esparcidos en una habitación (1/20). Si los hogares dejan el maíz en la mazorca (n=11), las ubicaciones de almacenamiento reportadas incluyeron un espacio de desván, techo o almacén en la casa (5/11), sacos o cajas (4/11) o estructuras de silo tradicionales (2/11). Si el maíz se compra en el mercado (n=18 que informaron), los lugares de almacenamiento informados incluyeron sacos (9/18), barriles (5/18) o silos (4/18). De los hogares que informaron cultivar su propio maíz y también comprar maíz en algún momento durante el año (n=18), solo el 11% (2/18) informó almacenar este maíz en un tipo diferente de lugar de almacenamiento en comparación con el maíz cultivado. Finalmente, el 97% de los hogares informaron que tienen la intención de mantener su maíz seco en el almacenamiento, pero las prácticas informadas variaron.

*Nixtamalización y consumo de maíz*

En cuanto a la preparación y el consumo de maíz por parte de los hogares, el 51% de los hogares informaron usar maíz amarillo, mientras que el 49% usa maíz blanco. Esta división uniforme generalmente se aplicó en todos los departamentos, excepto en Sacatepéquez, donde todos los hogares menos uno usaban maíz blanco. Se identificó una división uniforme similar entre los hogares que hacen y consumen tamales la mayoría del tiempo (51%) en comparación con tortillas (49%). Curiosamente, todos los hogares de Chimaltenango y Sacatepéquez informaron que preparan tortillas, mientras que el 81% (17/21) de los hogares de Quetzaltenango y Totonicapán informaron que preparan tamales. Todos los hogares preparan tortillas o tamales con maíz nixtamalizado.

Al preparar el maíz para la nixtamalización, ningún hogar rompe los granos de maíz antes de cocinarlo y solo dos hogares (6%) estarían dispuestos ha intentarlo. Para agregar la cal (hidróxido de calcio) al maíz, la mayoría de los hogares (91%) mezclan primero la cal y el agua, mientras que el 30% hierve la mezcla de agua y cal antes de agregarla al maíz. Todos los hogares informaron que utilizan una estufa de leña y dejan hervir el nixtamal, mientras que el 78,8% cubre el nixtamal para cocinar con una tapa. La Figura 2 y S1-S8 representan diagramas de caja de las diferentes proporciones de agua, cal y maíz, el tiempo de cocción y los tiempos de remojo en general y desglosados ​​por demografía. Curiosamente, en cuanto al tamaño de los hogares, los hogares más grandes (≥6 miembros) tuvieron tiempos de remojo más cortos y, al mismo tiempo, produjeron lotes más pequeños de tortillas o tamales. En los departamentos, los tiempos de cocción y de remojo fueron en general similares, salvo en los hogares de Chimaltenango, que tuvieron tiempos superiores al promedio y tamaños de lotes de tortillas o tamales inferiores al promedio (véanse las figuras 2a y 2c). Por último, hubo menos variación en los tiempos de cocción y de remojo, así como en los tamaños de lotes, entre los hogares que hablaban diferentes idiomas.

**Figura 2a-d**. Tiempos de cocción y de remojo por departamento y tamaño de hogar
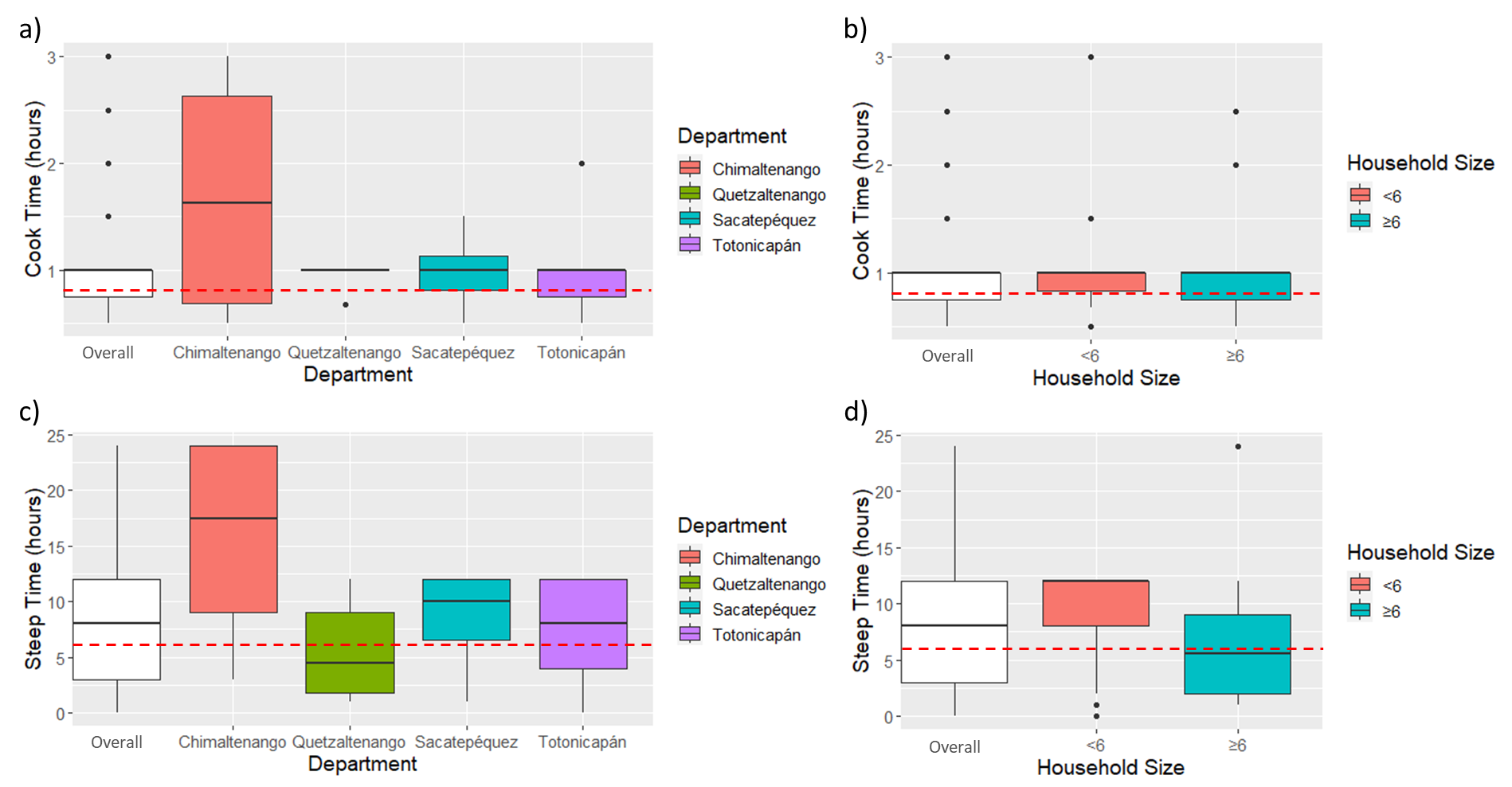


*Las líneas discontinuas indican el tiempo de cocción mínimo sugerido (40 minutos) y el tiempo de remojo mínimo sugerido (6 horas).*

El agua del grifo fue la fuente de agua más común utilizada para cocinar (78,8 %), y también se informó el agua de pozo (18 %) y el agua de tanque (3 %, n = 1). La mediana del número de veces que los hogares lavan el nixtamal después de remojarlo fue tres (rango de 0 a 7). El número de lavados varió de una mediana de dos entre los hogares de Sacatepéquez a una mediana de cuatro entre los hogares de Totonicapán. La mayoría de los hogares no reciclaron el agua de cocción (nejayote) ni el agua de lavado para ningún otro uso (84,8 %), mientras que se informó que regaron las plantas (n = 4) o los animales (n = 2). Todos los hogares informaron que usaban un molino comunitario para moler su maíz nixtamalizado y convertirlo en masa.

Finalmente, la media de tortillas o tamales consumidos por miembro del hogar por día fue de 6,6 (mediana de 5,7; rango: 0,6 – 16). La Figura 3 y las gráficas S9-S10 muestran diagramas de dispersión del consumo diario de tortillas o tamales por parte de los niños de los hogares. En general, a medida que los niños crecen, el consumo de tortillas o tamales también aumenta, pero hay una variación significativa. Curiosamente, los niños de los hogares de Totonicapán consumen muchas tortillas o tamales desde pequeños, mientras que la cantidad de tortillas o tamales que consumen los niños de Quetzaltenango sigue siendo baja a medida que los niños crecen. Por último, también se preguntó a los hogares sobre otros alimentos a base de maíz que preparan, que se enumeran en la Tabla 2. La mayoría de estos productos se elaboran con maíz nixtamalizado, excepto el pinol, que a menudo se elabora a partir de granos de maíz tostados y molidos.

**Figura 3**. Consumo diario de tortillas o tamales por parte de los niños (de 0 a 60 meses) entre los hogares
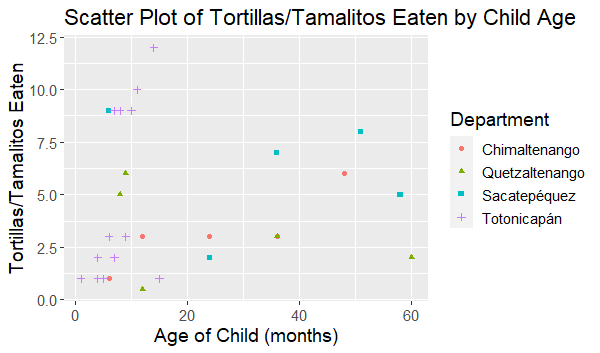


**Tabla 2.** Lista de otros productos a base de maíz que elaboran los hogares en Guatemala

| **Comida a base de maíz** | **Descripción** |
| --- | --- |
| Atol | Una bebida caliente tradicional hecha con masa de maíz, agua y, ocasionalmente, con sabor a canela, chocolate o fruta. |
| Agua de tortilla | Una bebida tradicional hecha con tortillas tostadas y agua, que a menudo se condimenta con sal o azúcar. |
| Agua de masa | Agua mezclada con masa de maíz disuelta, que se usa como base para sopas o bebidas, a veces condimentada o endulzada. |
| Tamales con recado o chuchitos | Los tamales son masa de maíz al vapor envuelta en hojas de mazorca (tusa), y "recado" hace referencia a un relleno de salsa sabrosa. Los chuchitos son una versión más simple y más pequeña, a menudo con una salsa a base de tomate y carne. |
| Tamalitos de chipilin o tayuyos | Tamales pequeños con sabor a chipilín, una hierba, o rellenos con una mezcla de ingredientes (como frijoles) envueltos en masa de maíz. |
| Maíz frito (tostadas, dobladas) | Platos de maíz frito; Las tostadas son tortillas planas y crujientes cubiertas con varios ingredientes, mientras que las dobladas son tortillas que se doblan y se rellenan. |
| Empanadas | Pastelitos rellenos hechos de masa y generalmente rellenos de carne, queso u otros ingredientes, que luego se fríen o se hornean. |
| Pinol | Polvo seco hecho a partir de granos de maíz tostado, que a menudo se mezcla con agua para preparar una bebida o se usa como ingrediente culinario. |

**Discusión**

*Prácticas poscosecha y almacenamiento del maíz*

Las prácticas de los pequeños productores en torno a la adquisición (cultivo y compra) del maíz, la poscosecha y el almacenamiento son fundamentales para controlar el crecimiento de hongos que pueden provocar la exposición a toxinas fúngicas. Garsow y sus colegas revisaron la literatura reciente sobre prácticas de precosecha y poscosecha del maíz asociadas con la contaminación por micotoxinas en el contexto guatemalteco (9). En el Altiplano Occidental, la cosecha suele tener lugar entre noviembre y diciembre; esto está respaldado por nuestros datos con algunos valores atípicos anteriores y posteriores. El secado del maíz después de la cosecha es fundamental para el control de hongos. Las oficinas de extensión del Departamento de Agricultura de los EE. UU. recomiendan un contenido máximo de humedad del maíz del 13 al 15 %, según el tiempo de almacenamiento planificado (13,14). Como los dispositivos de monitoreo son limitados en entornos similares a la ubicación de nuestra encuesta, maximizar el secado es fundamental. La literatura previa de Huehuetenango informó que el 93,5 % de los hogares secan su maíz antes del almacenamiento (15). Si bien nuestra encuesta no incluyó esta métrica específica, el 97% de los hogares sí informó que tenían la intención de mantener seco el maíz, y proporcionó ejemplos que incluían secarlo al sol y encontrar lugares secos para almacenarlo. Esto sugiere que los hogares demandan métodos para garantizar que el maíz se seque y almacene adecuadamente. Sin embargo, en ninguno de los estudios se evaluó la eficacia en el almacenamiento o en la reducción del contenido de humedad del maíz para cada uno de los métodos de secado utilizados por los hogares.

También recopilamos datos sobre qué proporción de hogares almacenaban el maíz todavía en la mazorca en comparación con el maíz desgranado (o descascarado) para almacenar solo los granos. Cada enfoque tiene implicaciones en las prácticas mejoradas para secar y almacenar el maíz. Nuestros datos sugieren que el 64% de los hogares desgranan o descascaran todo su maíz, mientras que el 36% conserva al menos una parte de la cosecha en forma de mazorca. Además, algunos hogares han informado que dejan las cáscaras en la mazorca del maíz sin desgranar (15). Es fundamental garantizar que exista una orientación clara y basada en datos para todos los enfoques, dada la amplia gama de recursos de secado y almacenamiento disponibles para los hogares. El maíz que se deja en la mazorca puede ayudar a mitigar la propagación de hongos si una mazorca está infectada, pero el secado completo puede ser más difícil y requerir tiempos de secado ligeramente más largos en comparación con el maíz desgranado. En el caso del maíz desgranado, los granos podridos deben seleccionarse y eliminarse para garantizar que no se propaguen los hongos. Finalmente, los datos anteriores sugieren que los sistemas de almacenamiento más comunes utilizados en Guatemala incluyen sacos, cajas de madera ("trojas"), colgadas del techo ("tapanco") o silos (16,17). Estos también se informaron comúnmente en nuestro estudio, pero también incluyeron barriles (principalmente de plástico). Es importante que, independientemente del sistema de almacenamiento, el maíz permanezca seco, la humedad se mantenga baja y, cuando sea posible, el sistema de almacenamiento promueva una aireación/ventilación adecuada para permitir temperaturas uniformes en todo el sistema (13). Los gradientes de temperatura pueden causar bolsas de humedad y acumulación, lo que genera áreas favorables para el crecimiento de hongos para el crecimiento de hongos. Curiosamente, de aquellos hogares que cultivaban y compraban maíz en algún momento del año, el 89% de ellos informaron que almacenaban el maíz en el mismo lugar. Esto puede ser un factor importante de contaminación cruzada si una fuente de maíz tiene mayor riesgo de contaminación por hongos (18).

Nuestros resultados y las pautas establecidas pueden ser útiles para desarrollar recursos y materiales de comunicación para los programas de extensión agrícola en Guatemala. Las métricas principales reportadas aquí parecieron ser consistentes en toda la geografía, lo que sugiere que es importante tener una variedad de materiales para apoyar a los hogares. Los hogares en Totonicapán parecieron tener una tasa más alta de no desgranar el maíz, pero se deben recopilar datos adicionales para verificar esta tendencia en una muestra más grande de la población. Comprender las prácticas de remojo del maíz en los hogares ayuda a los hogares a optimizar las prácticas para maximizar el control de la contaminación fúngica y reducir la exposición a toxinas.

*Procesos de nixtamalización*

Las prácticas de nixtamalización están menos estudiadas en Guatemala; sin embargo, los datos empíricos de los estudios de laboratorio sobre nixtamalización pueden brindar información sobre los rangos recomendados para los parámetros para maximizar la reducción de aflatoxina en el producto alimenticio a base de maíz resultante. Schaarschmidt y Fauhl-Hassek brindan una revisión de la nixtamalización en la reducción de aflatoxina en el maíz (10) y proporcionamos un conjunto general de umbrales para diferentes pasos en el proceso de nixtamalización basados ​​en esta revisión. En los estudios revisados, existe una amplia gama de efectividad reportada, dada la diversidad de contextos, cepas de maíz y hongos, y diseños de estudio. Sin embargo, los datos informados anteriormente sugieren que las variaciones en las concentraciones de cal utilizadas en el proceso pueden tener un efecto mínimo en los niveles de reducción de aflatoxina siempre que se alcance un umbral mínimo (sugerimos al menos 0,33 de relación cal-agua por masa). Sin embargo, los datos anecdóticos de nuestro proyecto sugirieron que los hogares suelen usar indicadores visuales para identificar la cantidad correcta de cal que se debe agregar. Traducir los valores umbral numéricos en indicadores visuales, si bien es difícil, podría beneficiar a los hogares.

En cuanto a los tiempos de cocción y remojo, los estudios revisados ​​por Schaarschmidt y Fauhl-Hassek 2019 sugirieron que un mínimo de 40 minutos de cocción y un mínimo de 6 horas de remojo son importantes para obtener reducciones de AFB1 superiores al 70%, pero los tiempos más largos generan mejores reducciones. En nuestra encuesta, el tiempo medio de cocción del nixtamal fue de una hora con n=4 hogares por debajo del mínimo recomendado de 40 minutos. El tiempo medio de remojo fue de ocho horas con n=8 hogares que informaron menos de 6 horas. Para aquellos hogares con más miembros, los tiempos de cocción y remojo fueron menores. Una mayor investigación sobre las prácticas de nixtamalización específicas de Guatemala ayudaría a las partes interesadas a desarrollar materiales de apoyo para que los hogares optimicen sus prácticas para la máxima reducción de toxinas fúngicas dada la viabilidad local y el contexto cultural.

En nuestro estudio, los principales productos alimenticios nixtamalizados fueron tortillas (principalmente en Chimaltenango y Sacatepéquez) o tamales (principalmente en Quetzaltenango y Totonicapán). Se plantea la hipótesis de que para preparar el maíz, romper los granos duros de maíz mejora la eficacia de la solución de cal (hidróxido de calcio) que penetra en el grano y reduce la aflatoxina; sin embargo, ningún hogar practica esta práctica y muy pocos hogares se interesaron en intentarlo.

Los hogares lavaron su maíz una media de tres veces. La literatura previa sugiere que el lavado es importante para ayudar a eliminar la aflatoxina del nixtamal, sin embargo, el lavado también reduce el pH del nixtamal, que es un mecanismo primario de desintoxicación de la aflatoxina (10). En nuestra encuesta, solo un hogar no lavó su nixtamal (solo escurrió el nejoyte) mientras que n=7 hogares lavaron su maíz más de 4 veces. La mayoría de los hogares también informaron que desecharon inmediatamente el nejoyte (agua de cocción) y el agua de lavado después de su uso, lo cual se recomienda ya que datos previos han demostrado que si la aflatoxina está presente en el maíz, se eliminará parcialmente a través de esta agua. Sin embargo, varios hogares lo utilizaron para regar las plantas o los animales. Finalmente, todos los hogares informaron que usaban un molino comunitario para moler el nixtamal en masa, sin embargo, no existen datos sobre la calidad y las prácticas de higiene de estos molinos. Datos adicionales sobre el riesgo de contaminación cruzada de toxinas fúngicas u otros contaminantes entre lotes de maíz podrían ayudar a identificar las prácticas mejoradas rentables para estos molinos comunitarios.

**Conclusión**

Este estudio piloto proporciona información a nivel de hogar sobre el manejo del maíz y las prácticas tradicionales de nixtamalización utilizadas por los hogares locales en diferentes entornos geográficos y culturales en Guatemala. Sin embargo, este estudio fue limitado en el tamaño de la muestra y la región geográfica dados los recursos disponibles y alentamos a que se trabaje más, especialmente en la comprensión de las prácticas locales de nixtamalización.

Los hallazgos resaltan conocimientos cruciales para los trabajadores de campo y los agentes de extensión agrícola que buscan mejorar las prácticas de manejo y nixtamalización del maíz en las zonas rurales de Guatemala. En primer lugar, la variabilidad en las prácticas de poscosecha y almacenamiento sugiere la necesidad de intervenciones localizadas y culturalmente sensibles que consideren las diferencias estacionales y geográficas. La implementación de programas educativos personalizados que enfaticen los beneficios de las técnicas adecuadas de secado y almacenamiento puede mitigar los riesgos de contaminación por aflatoxinas. En segundo lugar, la promoción de procesos optimizados de nixtamalización, como tiempos suficientes de cocción y remojo, puede mejorar la mitigación de la exposición. Si bien la mayoría de los hogares informaron que practican los pasos de nixtamalización en los umbrales informados anteriormente o por encima de ellos, es fundamental identificar enfoques sostenibles y económicos para que todos los hogares utilicen estas prácticas. Además, los servicios de extensión deben priorizar la difusión de conocimientos sobre el uso seguro del agua y la eliminación adecuada del nejayote para prevenir la exposición a las aflatoxinas. Abordar estas brechas puede empoderar a los hogares para que adopten métodos de procesamiento de maíz más seguros e informar el desarrollo de políticas y programas que salvaguarden la salud pública respetando a la vez las prácticas culturales.

**Agradecimientos**

Los autores agradecen a los dedicados coordinadores departamentales y a los agentes de extensión del Ministerio de Agricultura, Ganadería y Nutrición (MAGA) de Guatemala de los cuatro departamentos donde se realizó el estudio. Además, los autores agradecen a Bani Sandoval por su coordinación y orientación y a la Dra. Rosa Krajmalnik-Brown por sus comentarios y opiniones sobre el borrador inicial de este manuscrito. Esta investigación fue financiada por el Instituto Nacional de Ciencias de la Salud Ambiental, número de subvención 1R01ES033999-01A1. Las opiniones presentadas en este artículo son las de los autores y no representan las posiciones oficiales de las instituciones involucradas.

**Referencias**

1. Fung F, Wang HS, Menon S. Food safety in the 21st century. Biomedical Journal. 2018 Apr 1;41(2):88–95.

2. Fuentes Lopez MR, van Etten J, Ortega Aparicio A, Vivero Pol JL. Maíz para Guatemala: Propuesta para la Reactivación del Maíz Blanco y Amarillo. Guatemala, C.A.: FAO Guatemala; 2005.

3. Voth-Gaeddert LE, Torres O, Krajmalnik-Brown R, Rittmann BE, Oerther DB. Aflatoxin Exposure, Child Stunting, and Dysbiosis in the Intestinal Microbiome Among Children in Guatemala. Environmental Engineering. 2019;36(8):958–68.

4. Voth-Gaeddert LE, Stoker M, Torres O, Oerther DB. Association of aflatoxin exposure and height-for-age among young children in Guatemala. International Journal of Environmental Health Research. 2018;28(3):280–92.

5. Wild CP, Miller JD, Groopman JD. Mycotoxin Control in Low- and Middle- Income Countries. World Health Organization; 2016 p. 1–66.

6. Voth-Gaeddert LE, Stoker M, Torres OR, Oerther DB. The influence of local market and household factors on aflatoxin presence in maize and symptoms of its exposure to children in Guatemala. International Journal of Environmental Health Research. 2020;30(3).

7. Odjo S, Alakonya AE, Rosales-Nolasco A, Molina AL, Muñoz C, Palacios-Rojas N. Occurrence and postharvest strategies to help mitigate aflatoxins and fumonisins in maize and their co-exposure to consumers in Mexico and Central America. Food Control. 2022 Aug 1;138:108968.

8. Torres O, Matute J, Gelineau-van Waes J, Maddox JR, Gregory SG, Ashley-Koch a. E, et al. Human health implications from co-exposure to aflatoxins and fumonisins in maize-based foods in Latin America: Guatemala as a case study. World Mycotoxin Journal. 2015;8(2):143–59.

9. Garsow A, Mendez D, Torres O, Kowalcyk B. Evaluation of the impact of pre- and post-harvest maize handling practices on mycotoxin contamination on smallholder farms in Guatemala. World Mycotoxin Journal. 2022 May;15(3):261–8.

10. Schaarschmidt S, Fauhl-Hassek C. Mycotoxins during the Processes of Nixtamalization and Tortilla Production. Toxins. 2019 Apr;11(4):227.

11. Clave M. Actualización de la Perspectiva de Seguridad Alimentaria Mejoría en escenario de concretarse futura asistencia alimentaria Febrero 2015. FEWSNET. 2015;

12. MSPAS/INE/CDC. Encuesta Nacional de Salud Materno Infantil 2008 (ENSMI) 2008/2009. Guatemala: MSPAS/INE/CDC; 2010.

13. University of Minnesota Extension. Managing stored grain with aeration [Internet]. University of Minnesota; 2018 [cited 2024 Jun 20]. Available from: https://extension.umn.edu/corn-harvest/managing-stored-grain-aeration

14. Carlson CG, Reese CL. Grain Marketing - Understanding Corn Moisture Content, Shrinkage and Drying. SDSU Extension; 2019. Report No.: 35.

15. Mendoza JR, Sabillón L, Martinez W, Campabadal C, Hallen-Adams HE, Bianchini A. Traditional maize post-harvest management practices amongst smallholder farmers in Guatemala. Journal of Stored Products Research. 2017 Mar 1;71:14–21.

16. Gitonga ZM, De Groote H, Kassie M, Tefera T. Impact of metal silos on households’ maize storage, storage losses and food security: An application of a propensity score matching. Food Policy. 2013 Dec 1;43:44–55.

17. Mendoza JR, Rodas A, Oliva A, Sabillon L, Colmenares A, Clarke J, et al. Safety and Quality Assessment of Smallholder Farmers’ Maize in the Western Highlands of Guatemala. Journal of Food Protection. 2018;81(5):776–84.

18. Mendoza JR, Kok CR, Stratton J, Bianchini A, Hallen-Adams HE. Understanding the mycobiota of maize from the highlands of Guatemala, and implications for maize quality and safety. Crop Protection. 2017 Nov 1;101:5–11.
